# Simultaneous generation of first- to fourth-order OAM modes based on a cascaded preset-twist long-period fiber grating

**DOI:** 10.1515/nanoph-2024-0042

**Published:** 2024-04-15

**Authors:** Wenzhe Chang, Yan-ge Liu, Zekun Shi, Huiyi Guo, Xin Wang, Pan Wang, Zhi Wang

**Affiliations:** 12538 Institute of Modern Optics, Nankai University, Tianjin Key Laboratory of Micro-scale Optical Information Science and Technology, Tianjin 300350, China

**Keywords:** orbital angular momentum mode, long-period fiber grating, mode converter

## Abstract

We propose and demonstrate the simulation and fabrication of an all-fiber orbital angular momentum (OAM) mode converter capable of generating first- to fourth-order modes simultaneously, which is realized by inscribing a cascaded preset-twist long-period fiber grating (CPT-LPFG) in a six-mode fiber utilizing a CO_2_ laser. A new segmented Runge–Kutta method is proposed to simulate the preset-twist long-period fiber gratings. By calculating the twist angle and relative coupling coefficient for each pitch and then solving the coupled mode equations utilizing the Runge–Kutta algorithm. The simulation illustrates that the preset-twist method significantly improves the coupling coefficient of higher-order modes, thereby reducing coupling difficulty. In the experiment, by twisting the fiber at an angle of 1080° and fabricating cascaded gratings with periods of 745 μm, 310 μm, 204 μm, and 146 μm, it is feasible to generate first- to fourth-order OAM modes simultaneously, at wavelengths of 1635 nm, 1548 nm, 1460 nm, and 1334 nm, respectively. The insertion loss is less than 1 dB, and the mode purity is over 90 %. To the best of our knowledge, this is the first time that first- to fourth-order OAM modes are simultaneously generated utilizing a single long-period fiber grating.

## 1 Introduction

Since the discovery of the orbital angular momentum (OAM) of light by Allen et al. in 1992 [1], significant changes have occurred in the understanding and application of light. Vortex beams, which are characterized by the azimuthal factor exp(*ilφ*), carry OAM of *lh* per photon. Here, *l* is the topological charge (TC), *φ* represents the azimuthal angle, and *h* is Planck’s constant [2], [3]. OAM beams with a helical phase structure and donut-shaped intensity distribution are applicable in various fields, including optical communications [4–9], particle manipulation [10–13], imaging [14–16], remote sensing [17], [18], and quantum information processing [19]. Due to the property that OAM modes with different topological charges are orthogonal to each other, OAM mode division multiplexing (MDM) communication systems that employ few-mode fibers (FMFs) have the potential to significantly enhance the transmission capacity of optical communication systems [20–23]. A critical component of the MDM system is the mode converter, which can convert the fundamental mode to the OAM mode.

Several methods for generating OAM modes have been proposed and demonstrated. Methods based fiber include mode-selective couplers [24], [25], photonics lanterns [26], [27], and long-period fiber gratings (LPFGs) [28]. To date, there have been several fabrication methods for LPFGs, including UV exposure [29], mechanically induced micro-bending [30], CO_2_ laser inscribing [31], oxygen flame heating technology [32], etched technique [33], and femtosecond laser direct inscription [34], among which the LPFG inscribed by CO_2_ laser are extensively used for convenience. There is an increasing interest in the conversion of higher-order OAM modes, and the generation of first- to fourth-order OAM modes has been achieved in FMF using LPFGs [35]–[38]. Nonetheless, a significant limitation of most LPFGs is that they can only efficiently convert fundamental modes to a single higher-order mode, and the simultaneous generation of multiple OAM modes is extremely challenging. This leads to that a single mode converter can only convert a single OAM mode, and more channels require more mode converters. Therefore, there is an urgent need to explore the additional dimensions of OAM generation achieved by a single mode converter to improve transmission capacity.

It has been reported that multiple modes of the same order at different wavelengths can be generated simultaneously utilizing a single LPFG. For example, Wu et al. proposed a multi-channel OAM mode generation scheme based on an in-fiber mode selective interferometer (MSI), which can generate second-order modes at 17 wavelength channels [39]. Zhou et al. realized a multi-channel second-order OAM mode converter based on an elliptical-core helical intermediate-period fiber grating, and 10 wavelength channels were observed within the range of 1200 nm–2130 nm [40]. Jiang et al. proposed the inscription of parallel long-period gratings in a few-mode fiber using femtosecond lasers, achieving two and three channel third-order mode converter in a single FMF [34]. However, in FMF-based OAM mode division multiplexing communication systems, multiple channels of the same order modes are not enough to increase the channel capacity more efficiently, and realizing simultaneous generation of different higher-order modes on a single mode converter is still a challenge. Wang et al. realized the simultaneous generation of the first- and second-order OAM modes with conversion efficiencies of 94 % and 83 %, which was realized by using two consecutively cascaded helical long-period fiber gratings (HLPGs) [41]. In addition, they generated the second- and third-order OAM modes in a thinned four-mode fiber, using a common period to convert the fundamental mode to second-order mode first, and then convert the second-order to third-order mode [42]. This leads to significant crosstalk between modes, and the common resonance period of two modes is not universal. Jiang et al. proposed a mode converter realized by inscribing an HLPG using a femtosecond laser. By controlling the pitch of the HLPG, multiple higher-order OAM modes can be generated simultaneously [43]. Nonetheless, the generation of modes is not very flexible, and the wavelength and efficiency of each mode are difficult to control. In our previous work [44], we proposed a method for generating multiple higher-order OAM modes in a single LPFG by utilizing a high diffraction order of gratings. However, an accurate simulation of the preset twist method is lacking, and flexible control of the resonant wavelength of each higher-order mode is not achievable in the experiment. In other words, changing the resonant wavelength of one mode has an impact on the other modes, which is a significant limitation for multimode converters. Therefore, there is still an urgent need for a general and flexible method to achieve simultaneous mode conversion between different higher-order modes on a single device.

In this paper, we demonstrate a highly compact mode converter in a six-mode fiber inscribed by a CO_2_ laser using a cascaded preset twist LPFG for simultaneous conversion of fundamental mode to first- to fourth-order mode at different wavelengths. A segmented Runge–Kutta method is proposed to simulate the preset twist gratings, where the twist angles and relative coupling coefficients are calculated for each pitch, and then the coupled mode equations are solved approximately utilizing the Runge–Kutta algorithm. The simulation demonstrates that the preset-twist method can effectively improve the coupling coefficients of the higher-order modes, consequently reducing the difficulty of coupling, with the best effect being achieved by using a twist angle of 1080°. In the experiments, by applying a preset twist angle of 1080° to the fiber and employing cascade gratings with periods of 745 μm, 310 μm, 204 μm, and 146 μm, first- to fourth-order OAM modes are simultaneously generated in a six-mode fiber at wavelengths of 1635 nm, 1548 nm, 1460 nm, and 1334 nm, respectively. The conversion efficiencies are higher than 97 %, and the mode purities are measured to be higher than 90 % with an insertion loss of less than 1 dB.

## 2 Operation principle and theoretical analysis

**Figure 1:**
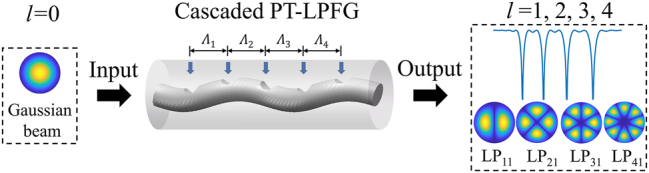
Concept of simultaneous generation of first- to fourth-order OAM modes using a CPT-LPFG.

The concept of using a cascaded preset twisted long-period fiber grating (CPT-LPFG) to achieve the simultaneous generation of first- to fourth-order OAM modes is shown in Figure 1. When a Gaussian beam is incident into the CPT-LPFG, the output beams are different order modes at different wavelengths, which is achieved by cascading four gratings with different periods Λ. In order to generate the first- to fourth-order modes, the fiber used in the simulation and experiment is a step index six-mode fiber (6 MF, Yangtze Optical Fiber and Cable, FM2012-B). The core and cladding diameters are 16 μm and 125 μm, respectively, and the relative refractive index difference between core and cladding is 0.75 %, which can support the transmission of LP_11_, LP_21_, and LP_31_ modes at 1200–1700 nm and LP_41_ mode at 1200–1400 nm. Here, the grating periods Λ can be obtained by the phase matching condition shown in Eq. (1) [45]

(1) $$\beta_{0}-\beta_{l}-\frac{2\pi }{\Lambda_{l}}=0$$

where *β*_0_ and *β*_*l*_ are the propagation constants of the fundamental mode and the *l*-order modes, respectively. According to Eq. (1), the grating periods for the conversion from the fundamental mode to the first- to fourth-order modes at 1600 nm, 1500 nm, 1400 nm, and 1300 nm are 732 μm, 334 μm, 209 μm, and 148 μm, respectively. When the phase matching conditions of the fundamental mode and the first- to fourth-order modes are satisfied simultaneously, the resonant coupling occurs at the corresponding four different wavelengths, respectively. In addition, the resonant wavelength of the mode coupling is closely related to the grating period, and the couplings for the four higher-order modes are completely independent, so that the resonant wavelengths of the first- to fourth-order modes can be flexibly controlled by changing the grating periods.

Although the coupling of different order modes can be realized simultaneously by cascading gratings, generating higher-order modes remains challenging. As the fundamental mode is converted to a higher-order mode, the effective refractive index difference increases and the cross-coupling coefficient decreases with the increase of mode order, both of which result in a stronger refractive index modulation being required for the generation of higher-order modes. However, it is important to consider that a large insertion loss may be introduced due to a strong refractive index modulation with the single-exposure CO_2_ laser manufacturing method, which is a significant issue for cascaded gratings. Therefore, in our previous work, we proposed a preset-twist method for generating higher-order modes by introducing angular refractive index modulation and effectively generated second- to fourth-order OAM modes in experiments [38], [46], [47]. Nevertheless, there has been a lack of accurate simulation of the preset-twist LPFGs, and there is an urgent need to propose an accurate method to simulate the change of angular refractive index modulation to better guide the experiments.

**Figure 2:**
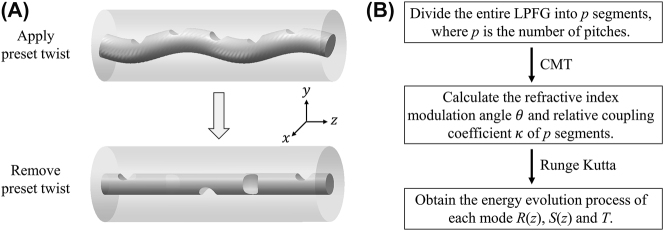
Schematic diagram and simulation method of CPT-LPFG. (A) Schematic diagram of CPT-LPFG when applying and removing the preset twist. (B) Flow chart of our segmented Runge–Kutta method for the CPT-LPFG.

**Figure 3:**
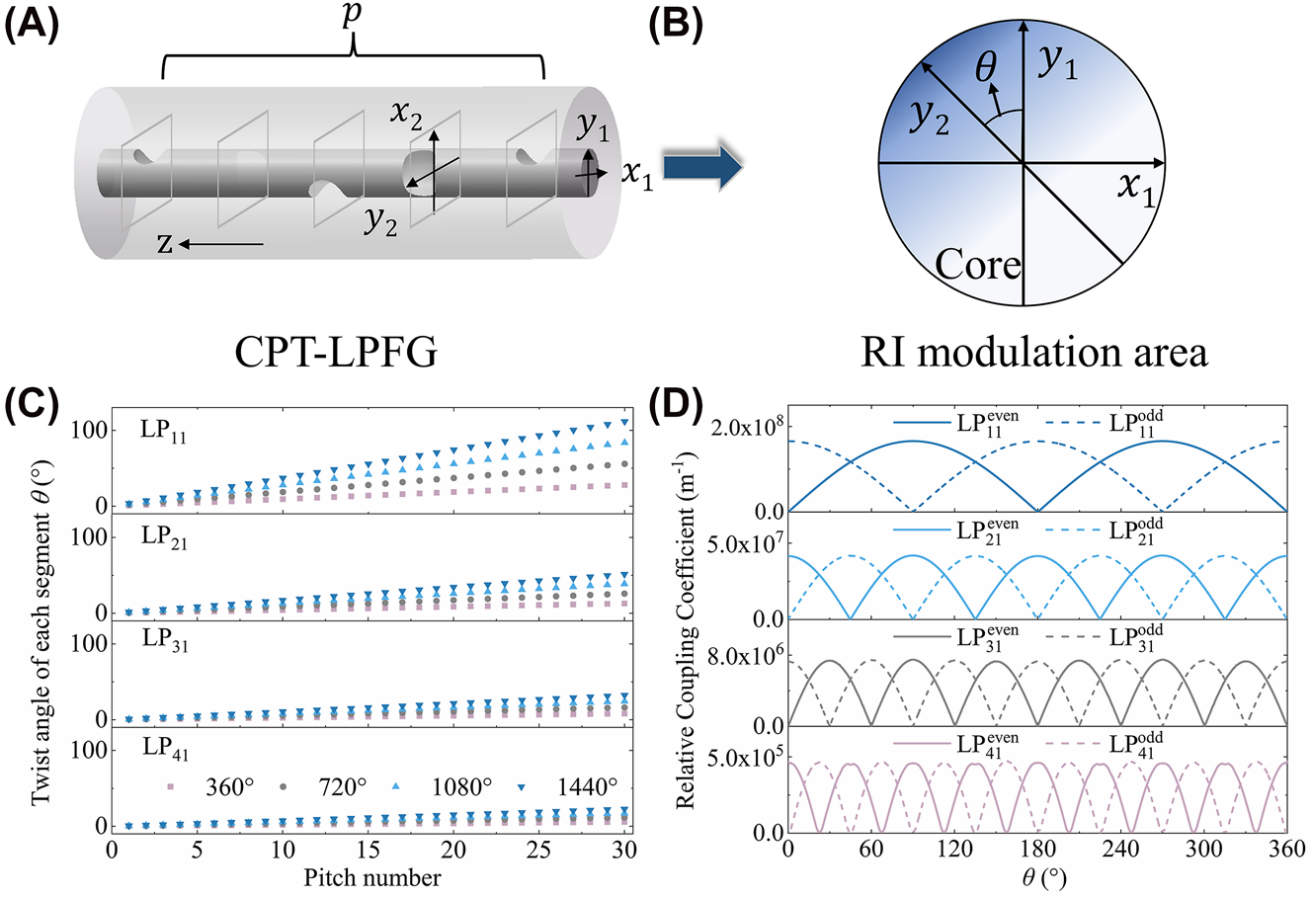
Twist angles and coupling coefficients for each segment obtained by the segmented Runge–Kutta method. (A) Schematic diagram of CPT-LPFG divided into *p* cross sections according to the pitch number. (B) Refractive index modulation area of each segment when the modulation direction is *θ*, for example. (C) Twist angle of each segment when applying different twist angle to the fiber for first- to fourth-order mode. (D) The relative coupling coefficients between the fundamental mode and the first- to fourth-order modes when the twist angle *θ* is increased from 0 to 360°.

Here, we propose the segmented Runge–Kutta method to simulate the cascaded preset-twist LPFGs. The schematic diagram of CPT-LPFGs for applying and removing the preset twist and the flow chart of the segmented Runge–Kutta method are shown in Figure 2. The first step is segmentation. The CPT-LPFG is partitioned into *p* segments based on the pitch number and approximated that each segment is uniform. When the length of each segment is short enough and the solving accuracy is high enough, the overall accuracy can be well guaranteed. Secondly, the refractive index modulation angle of each segment is calculated, and then the relative coupling coefficients at each segment are solved using coupled mode theory. As depicted in Figure 2A, when a certain angle of preset twist is applied to the fiber before exposure, and the refractive index modulation is periodically applied to the fiber at this time, the direction of the refractive index modulation in each segment aligns with the *y*-axis direction of the fiber. Then, when the preset twist is removed after exposure, there is no significant change in the coupling efficiency of the higher-order modes, which indicates that the refractive-index modulation caused to the fiber is permanent. However, the direction of the refractive index modulation at each segment changed significantly, where the direction of the refractive index modulation at each segment is at a regular angle to the direction of the *y*-axis of the fiber. As illustrated in Figure 3A and B, the initial axes of fiber cross section are defined as the *x*_1_ and *y*_1_ axes. Then, *p* cross sections are taken along the *z*-direction of the fiber propagation, where *p* is the pitch number or the number of segments divided in the first step. At each cross section, the direction of refractive index modulation is different and defined as *y*_2_, where the angle between *y*_2_ and *y*_1_ is *θ*. If a total twist angle of *β* is applied to the grating of length *L*, and the grating periods is Λ, then the twist angle of each segment *θ* is $\frac{\beta }{L}\cdot \Lambda$ . For the total twist angles of 360°, 720°, 1080°, and 1440° applied to the grating of length *L*, the twist angles of each segment are calculated as shown in Figure 3C for the first- to fourth-order modes. It can be found that as the mode order increases, the periods become shorter and the twist angle at each segment is smaller. In addition, the twist angle at each segment increases multiplicatively as the overall twist angle increases.

After calculating the refractive index modulation angle of each segment, the coupling coefficients of each segment are solved using coupled mode theory. To assess the impact of varying angles on the relative coupling coefficients, as illustrated in Figure 3D, the relative coupling coefficients between the fundamental mode and the first- to fourth-order modes when the angle is increased from 0 to 360° are calculated employing Eq. (2), respectively, where the even mode represented by the solid line and the odd mode as the dashed line.

(2) $$\kappa_{\mu \nu }\left(z\right)\approx \frac{\omega }{4}\iint E_{\mu }^{*}\left(x,y\right)\Delta \varepsilon \left(x,y\right)E_{\nu }\left(x,y\right)dxdy$$

where $\kappa_{\mu \nu }\left(z\right)$ is the coupling coefficient between the *μ* mode and *ν* mode, *ω* represents the circular frequency, $E_{\mu }\left(x,y\right)$ and $E_{\nu }\left(x,y\right)$ represent the electric field distribution of the *μ* mode and *ν* mode, and $\Delta \varepsilon \left(x,y\right)$ represents the perturbation to the permittivity caused by the perturbation of the refractive index [45], [48], [49]. In addition, when the grating is fabricated utilizing the exposure of CO_2_ laser manufacturing method, due to the thermal diffusion effect, the refractive index modulation area of each segment has an exponentially decreasing distribution from outside to inside along the *y*_2_-axis direction of the modulation angle here, and the refractive index modulation Δ*n* can be expressed as [50]

(3) $$\Delta n\left(x,y\right)=\left\{\begin{array}{cc} \Delta n_{\max}e^{-\alpha \left(r-mc-\sqrt{x^{2}+{\left(y-mc\right)}^{2}}\right)} & \left(\sqrt{x^{2}+y^{2}\leq r}\right)\\ 0 & \left(\text{others}\right) \end{array}\right.$$

where Δ*n*_max_ is the maximum refractive index modulation, which is related to the CO_2_ laser exposure power, and *α* and *r* are the attenuation factor and the core radius of the fiber. *mc* is a certain value that affects the contour curvature of the refractive index modulation area, and it is close to the actual situation when it is taken as −40 according to VanWiggeren et al. [51]. It is worth mentioning that the coupling coefficient is related to the maximum refractive index modulation that is artificially defined. The relative coupling coefficients are calculated by normalizing the maximum refractive index modulation to exclude the interference term of the customized maximum refractive index modulation. The analysis reveals that the coupling coefficients still decrease with the increase of mode order but vary significantly with the change of twist angle. In addition, if the mode order is *l*, the number of cycles for the coupling coefficients of even and odd modes is 2*l* as the angle increases from 0° to 360°. Furthermore, the coupling coefficients of the odd and even modes alternate, with the odd mode reaching its maximum value at an angle of $\frac{\pi }{2l}\times \frac{{\left(-1\right)}^{l}+1}{2}+\frac{k}{l}\pi$ and the coupling coefficient of the even mode having a minimum value. Conversely, with the even mode obtaining a maximum value at an angle of $\frac{\pi }{2l}\times \frac{{\left(-1\right)}^{l+1}+1}{2}+\frac{k}{l}\pi$ and the coupling coefficient of the odd mode reaches its minimum value. Therefore, after obtaining the angle of the refractive index modulation area of each segment from Figure 3C, the corresponding relative coupling coefficients for each segment can be calculated as illustrated in Figure 3D.

Finally, the coupled mode equations are solved using the Runge–Kutta method. To obtain the evolution of the energy for each mode in the fiber throughout the propagation along the LPFG, it is necessary to solve the coupled mode equations, which can be transformed into a linear differential equation system that is presented in Eq. (4) [45].

(4a) $$\frac{\text{d}R\left(z\right)}{dz}=i\bar{\sigma }R\left(z\right)+i\kappa_{ac}S\left(z\right)$$

(4b) $$\frac{\text{d}S\left(z\right)}{dz}=-i\bar{\sigma }S\left(z\right)+i\kappa_{ac}R\left(z\right)$$

where the amplitudes are $R\left(z\right)=\cos\left(\gamma z\right)+i\frac{\bar{\sigma }}{r}\sin\left(\gamma z\right)$ and $S\left(z\right)=i\frac{\kappa_{ac}}{r}\sin\left(\gamma z\right)$ . In these equations, *z* is the direction of light propagation along the fiber, *R*(*z*) and *S*(*z*) are the amplitude of the fundamental mode and the amplitude of the higher-order mode along the *z* direction, respectively, $\bar{\sigma }$ is the general self-coupling coefficient, $\gamma^{2}=\sqrt{\kappa_{ac}^{2}+{\bar{\sigma }}^{2}}$ , and *κ*_*ac*_ is the cross-coupling coefficient. For uniform LPFGs, the solutions can be used directly to calculate mode energies at the output. However, for nonuniform LPFGs like the proposed CPT-LPFG, the Runge–Kutta method is required to solve the coupled mode equations.

**Figure 4:**
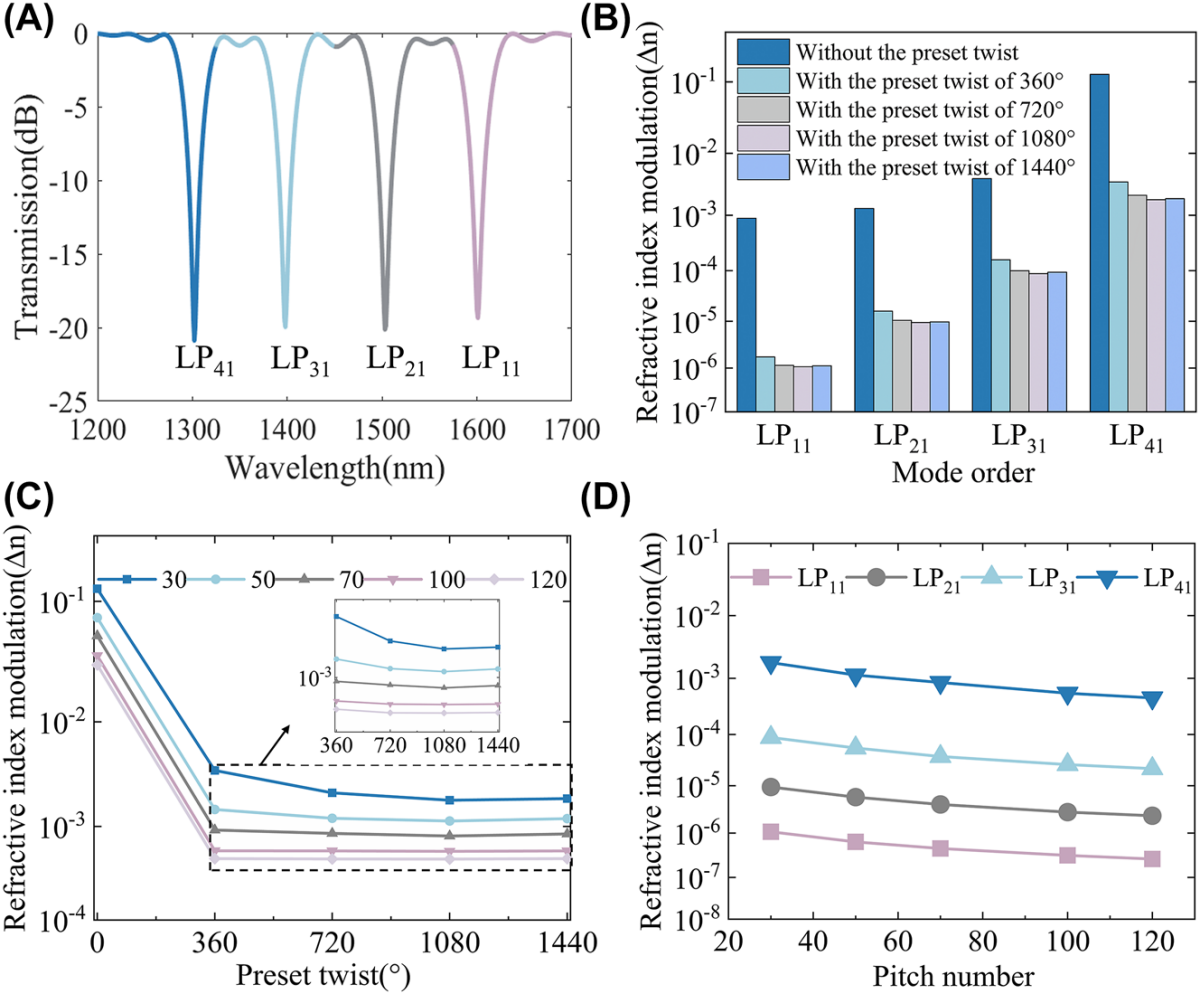
The simulated transmission spectrum and the required refractive index modulation for different modes with different twist angles and pitch numbers. (A) The transmission spectrum for simultaneous generation of the fundamental mode to first- to fourth-order modes when the coupling efficiency is 99 %. (B) The refractive index modulation required to achieve 99 % coupling efficiency when applying twist angles of 0°, 360°, 720°, 1080°, and 1440° with the pitch number of 30. (C) The refractive index modulation required to achieve 99 % coupling efficiency for twist angles of 0°, 360°, 720°, 1080°, and 1440° with different pitch numbers of 30, 50, 70, 100, and 120 for the fourth-order mode. (D) The refractive index modulation required to achieve 99 % coupling efficiency for the first- to fourth-order modes with different pitch numbers.

To simulate the entire grating, it is necessary to superimpose each segment. The initial conditions for the first segment are *R*(0) = 1 and *S*(0) = 0, which represent the mode incident to the first grating as fundamental modes. For all subsequent segments, the initial conditions are the solutions of the preceding segment. After solving the last segment, the evolution of mode energies *R*(*z*) and *S*(*z*) as they propagate along the LPFG for a length of *L* can be determined, and thus the transmission spectrum can be calculated. Figure 4A depicts the transmission spectra of the fundamental mode to the first-, second-, third-, and fourth-order modes being simultaneously generated, with a coupling efficiency of 99 %; in other words, the depth of the transmission spectrum is 20 dB. To compare the impact of varying twist angles on fabricating gratings, the required refractive index modulation to achieve 99 % coupling efficiency is calculated in Figure 4B for twist angles of 0°, 360°, 720°, 1080°, and 1440° with pitch number of 30. The assumptions made in this calculation are that the fundamental modes are equally converted into even and odd modes. For the first-order mode, the refractive index modulation required to achieve a 99 % coupling efficiency at twist angles of 0°, 360°, 720°, 1080°, and 1440° is 8.86 × 10^−4^, 1.75 × 10^−6^, 1.15 × 10^−6^, 1.07 × 10^−6^, and 1.12 × 10^−6^. For the second-order mode, the required refractive index modulation is 1.30 × 10^−3^, 1.61 × 10^−5^, 1.04 × 10^−5^, 9.40 × 10^−6^, and 9.70 × 10^−6^, while for the third-order mode, the needed modulation is 4.03 × 10^−3^, 1.60 × 10^−4^, 9.96 × 10^−5^, 8.78 × 10^−5^, and 9.30 × 10^−5^, and for the fourth-order mode, modulation of 1.25 × 10^−1^, 3.56 × 10^−3^, 2.16 × 10^−3^, 1.83 × 10^−3^, and 1.90 × 10^−3^ is necessary. Applying a preset twist to the grating effectively reduces the refractive index modulation required to achieve the same coupling efficiency. In other words, the coupling difficulty of higher-order modes is reduced. In addition, it can be found in Figure 4B that 1080° is the most effective angle for the generation of first- to fourth-order modes with the same pitch number of 30. To eliminate the effect of pitch number, take the fourth-order mode as an example, we simulated the refractive index modulation required to achieve 99 % coupling efficiency for twist angles of 0°, 360°, 720°, 1080°, and 1440° with different pitch numbers of 30, 50, 70, 100, and 120 as shown in Figure 4C. It shows that a preset twist angle of 1080° is the most effective in reducing the coupling difficulty of higher-order modes. Therefore, the preset twist angle is set to 1080°, and the refractive index modulation required to achieve 99 % coupling efficiency for the first- to fourth-order modes with different pitch number is simulated as shown in Figure 4D. The results indicate that the increasing of pitch number can also effectively reduce the coupling difficulty of the higher-order modes with the same twist angle. In conclusion, this provides an effective guide to the design of experiments, which works best at a preset twist angle of 1080°, and in order to reduce the differences of coupling difficulty between modes of different orders, it can be achieved by increasing the exposure power and increasing the pitch number for the higher-order modes in the experiments.

## 3 Experimental results and discussions

**Figure 5:**
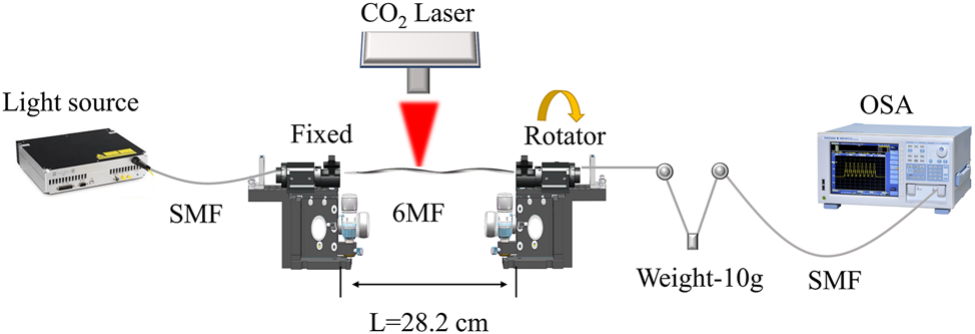
Schematic diagram of the experimental setup.

**Figure 6:**
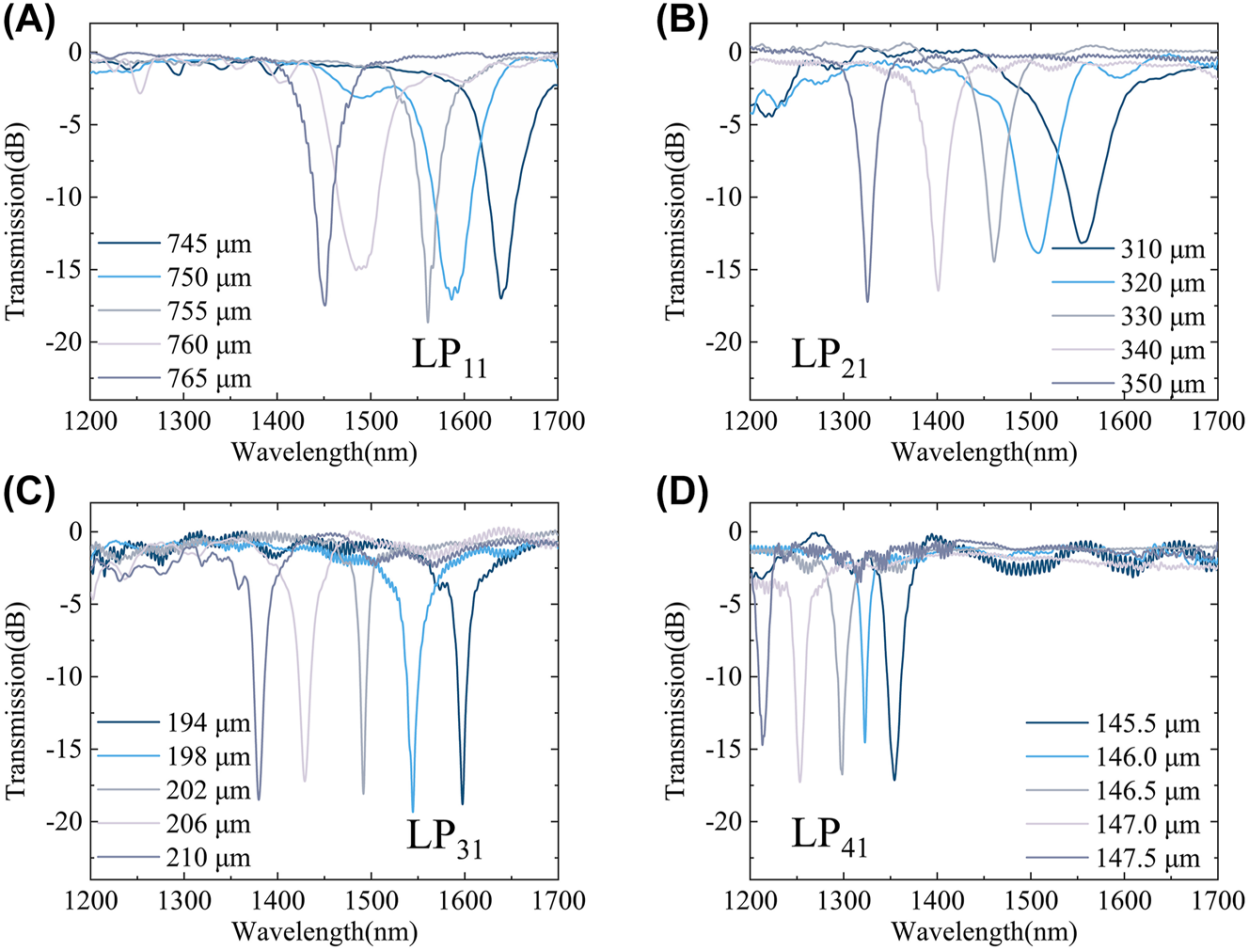
The relationship between the resonant wavelengths and the grating periods of (A)–(D) the first- to fourth-order modes.

In the experiment, the conversion from fundamental mode to first-, second-, third-, and fourth-order modes was achieved in a six-mode fiber using a CO_2_ laser (CO_2_–H30, Han’s laser), and the experimental setup for the fabrication of LPFG is shown in Figure 5. A section of six-mode fiber was clamped on both sides with a clamping table of length 28.2 cm. Before exposure, the fiber was twisted at an angle by gradually rotating the right rotating platform while fixing the left clamping table. Subsequently, one side of the 6 MF was irradiated with a CO_2_ laser from above. The periods of the fundamental mode coupled to the first-, second-, and third-order modes at 1550 nm are set to 755 μm, 310 μm, and 198 μm, respectively, and the period of the fundamental mode coupled to the fourth-order mode at 1310 nm is set to 146 μm, all of which correspond to the first diffraction order to prevent any crosstalk with other modes. The total twist angles applied to the fibers are all 1080° and since the distance between the rotator and the clamp is 28.2 cm, so the preset twist per unit length is 38.29°/cm. The preset-twist LPFGs converted from fundamental mode to first- to fourth-order modes can be fabricated using powers of 2.10 W, 2.25 W, 2.40 W, and 2.80 W, with pitch numbers of 30, 50, 100, and 120, respectively. Additionally, to flexibly control the resonant wavelength of mode coupling, the relationship between the resonant wavelengths and the grating periods of the first- to fourth-order modes was investigated as shown in Figure 6. The transmission spectra were observed using a supercontinuum source (YSL photonics SC-5) and an optical spectrum analyzer (OSA, AQ6375). The resonant wavelengths of all four modes are inversely proportional to the grating periods, and mode coupling can be achieved at different wavelengths when the grating period is changed.

**Figure 7:**
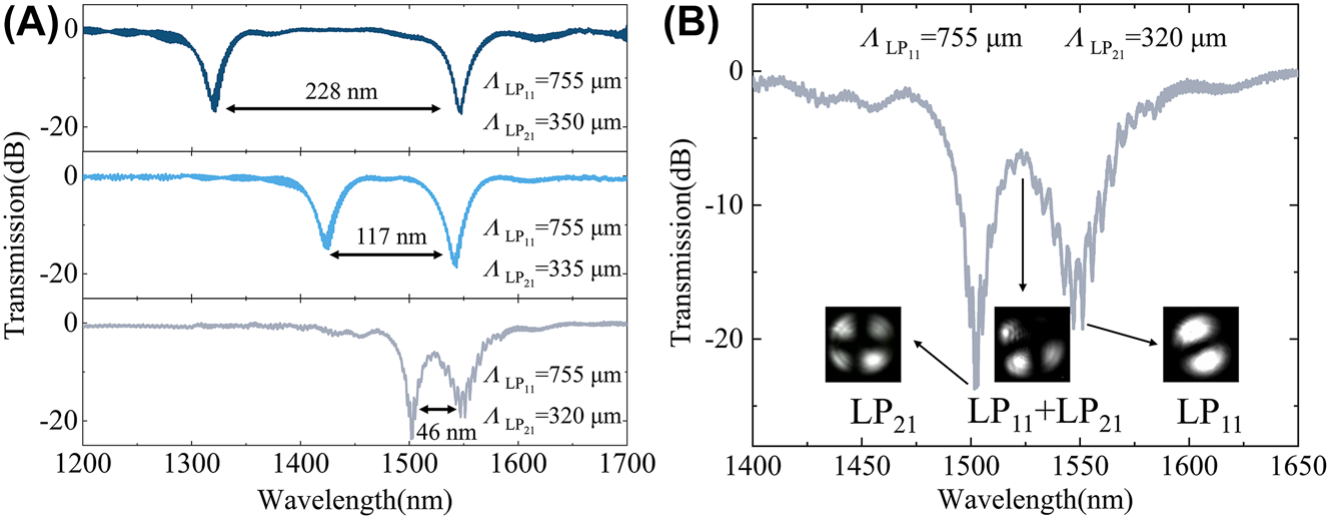
Adjust the resonance wavelength and wavelength interval freely, and generate pure and mixed modes flexibly using CPT-LPFG. (A) The transmission spectrum for the CPT-LPFG with simultaneous generation of first- and second-order modes when the first grating period was set to 755 μm and the second grating period was set to 350 μm, 335 μm, and 320 μm, respectively. (B) The mode profiles of three typical wavelengths, where high purity second-order modes and first-order modes can be observed at the resonant wavelength of 1501 nm and 1547 nm, while mixed modes of the first and second orders are generated at 1524 nm.

The resonant wavelength of each mode and the wavelength interval between modes can be freely adjusted and flexibly controlled. As shown in Figure 7A, taking the CPT-LPFG for generating first-order and second-order modes as an example. In the experiment, a preset twist of 38.29°/cm was applied to the fiber before exposure. The first grating period was set to 755 μm, with a pitch number of 30, to achieve the mode coupling between fundamental mode and first-order mode at 1547 nm under a power of 2.10 W. For the second grating, the period was set to 350 μm, 335 μm, and 320 μm with the pitch number of 50, and the mode coupling of the fundamental mode to the second-order mode was realized at 1319 nm, 1430 nm, and 1501 nm under a power of 2.25 W, respectively. As the grating period of the second-order mode decreases, the resonant wavelength gradually increases, and thus the wavelength interval between the two modes decreases. The corresponding wavelength intervals are 228 nm, 117 nm, and 46 nm, respectively. As a result, mode coupling at any wavelengths can be achieved simply by adjusting the grating periods. Similarly, the resonant wavelength and wavelength interval can also be flexibly adjusted for any other modes.

**Figure 8:**
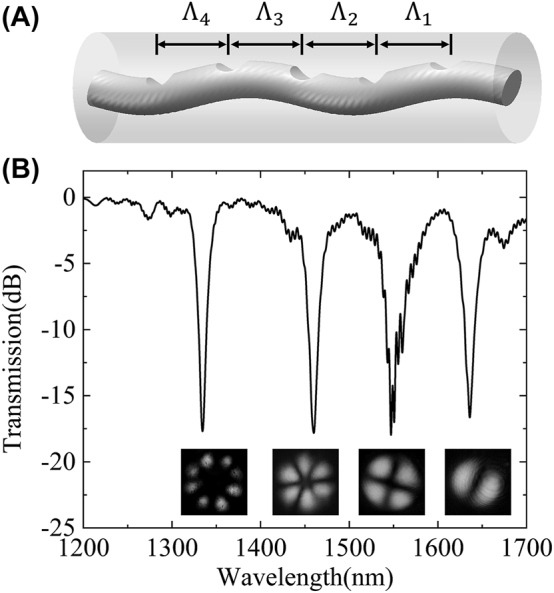
The CPT-LPFGs for the simultaneous generation of first- to fourth-order modes. (A) Structure of final CPT-LPFG and (B) the transmission spectrum and mode distributions.

Furthermore, the CPT-LPFG has the capability to flexibly generate both pure and mixed modes. On the one hand, if the wavelength interval between the two modes is significant, the purity of both modes generated at the resonant wavelength is high enough for the generation of pure modes. For instance, in the upper two subplots of Figure 7A, when the periods of the first-order and second-order modes are 755 μm, 350 μm and 755 μm, 335 μm, respectively, pure first-order and second-order modes generated at the resonant wavelengths of 1319 nm, 1547 nm, and 1430 nm, 1547 nm, while the fundamental mode or a single order mode with low purity are generated at other wavelengths. On the other hand, if there is a small wavelength interval between the two modes, it is possible to generate both pure and mixed modes. For example, when the periods of the first-order and second-order modes are 755 μm and 320 μm, respectively, the modes generated at the resonant wavelengths of 1501 nm and 1547 nm are both pure modes. However, the modes generated between the resonance wavelengths are mixed modes of both the first-order and second-order modes. Figure 7B illustrates the mode profiles of three typical wavelengths, including 1501 nm, 1524 nm, and 1547 nm, observed using the setup in Figure 8. High-purity second-order mode and first-order mode are observed at the resonant wavelength of 1501 nm and 1547 nm, respectively, when the resonant wavelengths overlap, such as at 1524 nm, mixed modes of the first- and second-order modes are generated.

After solving the coupling issues, the CPT-LPFG can be used to achieve the simultaneous generation of different order modes flexibly, where the mode order and resonant wavelength can be controlled through different grating periods. As illustrated in Figure 8A, to achieve the simultaneous generation of first- to fourth-order modes, the final CPT-LPFG is a cascade of four preset-twist LPFGs. Specifically, a preset twist is applied to the fiber before exposure using a CO_2_ laser, where Λ_1_, Λ_2_, Λ_3_, and Λ_4_ correspond to the periods of coupling from the fundamental mode to the first- to fourth-order modes, respectively. In the experiment, the periods of the first-order diffraction order are utilized for all modes to prevent crosstalk from other modes, resulting in periods of 745 μm, 310 μm, 204 μm, and 146 μm for the fundamental mode coupled to the first- to fourth-order modes, respectively. The pitch numbers are 30, 50, 100, and 120, and the powers are 2.10 W, 2.25 W, 2.40 W and 2.80 W, respectively. In addition, to fabricate the four preset-twist LPFGs simultaneously and achieve mode coupling most easily, a twist angle of 1080° is utilized for all of them. Eventually, the CPT-LPFG with simultaneous generation of first- to fourth-order modes was achieved, and the transmission spectrum was shown in Figure 8B, where the couplings of the fundamental mode to the first- to fourth-order modes were achieved simultaneously at 1635 nm, 1548 nm, 1460 nm, and 1334 nm, respectively. The conversion efficiencies of the four modes are 97.87 %, 97.56 %, 98.33 %, and 98.24 %, respectively, corresponding to the resonant dip in spectrum ∼−16.66 dB, ∼−16.09 dB, ∼−17.75 dB, and ∼−17.58 dB. Moreover, the measured insertion losses of the four modes are 0.38 dB, 0.50 dB, 0.56 dB, and 0.91 dB, which are all less than 1 dB. The results demonstrate that the simultaneous generation with high efficiency and low loss can be achieved at the same time for the four angular modes supported by the six-mode fiber, and the resonant wavelength can be flexibly controlled through the grating periods. It is worth mentioning that since the six-mode fiber only supports the transmission of four angular modes, four different modes were generated simultaneously in the experiment. Theoretically, the method can be used to generate more modes of different orders simultaneously if supported.

**Figure 9:**
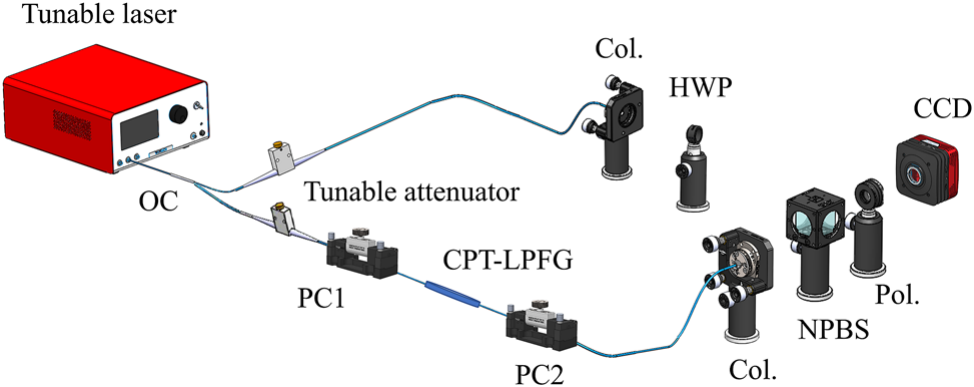
Experimental setup for the generation and detection of OAM modes. OC, optical coupler; PC, polarization controller; CPT-LPFG, cascaded preset-twist long period fiber grating; Col., collimator; NPBS, nonpolarization beam splitter; Pol., polarizer; HWP, half-wave plate; CCD, charge-coupled device.

**Figure 10:**
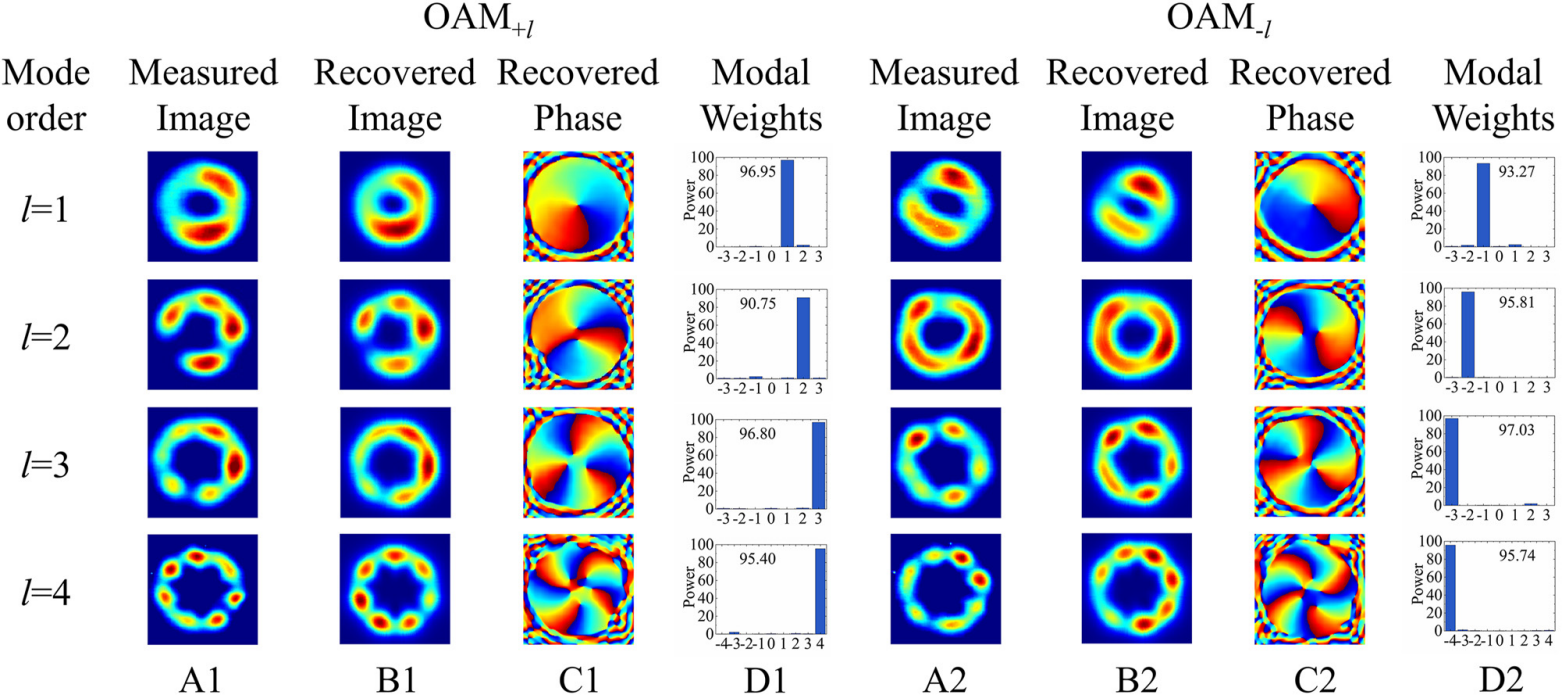
The output mode intensity distributions of CPT-LPFG and the results of OAM mode measurements. (A) The measured intensity distributions, (B) the recovered intensity distributions, (C) the recovered phase patterns, and (D) the modal weights and mode purities for the OAM_±1_, OAM_±2_, OAM_±3_, and OAM_±4_ modes.

The experimental setup utilized to generate OAM modes and measure the mode purity is illustrated in Figure 9. The output beam from the tunable lasers (EXFO T100S-HP, 1260 nm–1360 nm or KEYSIGHT 81600B, 1460 nm–1640 nm) is divided into two branches by an optical coupler with a ratio of 5:5. The lower branch generates OAM beams while the upper branch serves as a reference beam, interfering with the generated OAM beams to determine the mode order and measure the more purity. Among them, two tunable attenuators are utilized to adjust the optical power to ensure the contrast of the final patterns. Specifically, in the lower branch, the LP_01_ mode is converted to the LP_11_, LP_21_, LP_31_, and LP_41_ modes by the CPT-LPFG. By adjusting the polarization controllers (PCs) before and after the CPT-LPFG, a phase difference of π/2 is introduced, and the conversion of linearly polarized modes to OAM modes follows the principle of OAM_±*l*_ = ${\text{LP}}_{l1}^{\text{even}}$ ± *i* ${\text{LP}}_{l1}^{\text{odd}}$ [31]. In the upper branch, a half-wave plate (HWP) is utilized to change the polarization direction of beam to avoid extinction. At last, a nonpolarization beam splitter (NPBS) is employed to combine the beams from both branches. After passing through the NPBS, a polarizer is established to obtain the information of higher-order modes at each polarization. The final patterns of first- to fourth-order OAM modes are captured by a charge-couple device (CCD, Xi’an leading optoelectronic, LD-SW640171550-UC-G) as shown in Figure 10. By adjusting the wavelength of the laser and the polarization controller, different orders of OAM modes with donut shapes can be observed, and the intensity distributions of the generated OAM modes at four wavelengths are recorded.

**Figure 11:**
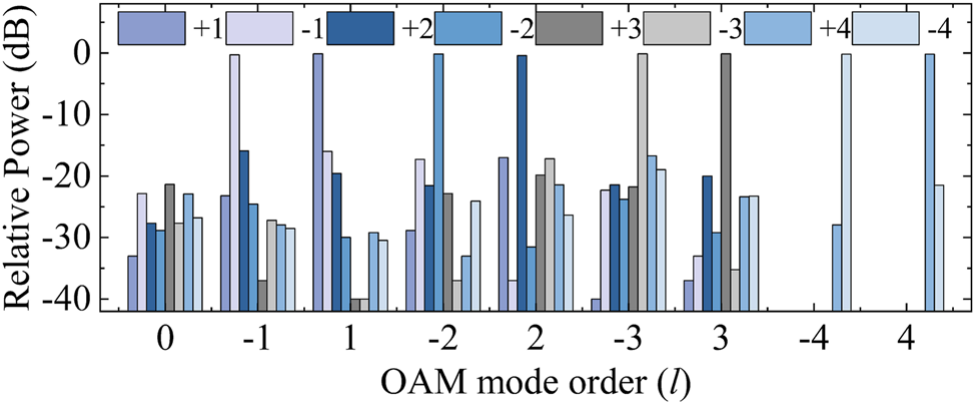
The power ratios for each component of the OAM_±1_, OAM_±2_, OAM_±3_, and OAM_±4_ modes at 1635 nm, 1548 nm, 1460 nm, and 1334 nm waveband.

To evaluate the quality of the modes, the purity of the generated OAM modes was measured through a fast modal decomposition for fibers using digital holography [52], [53]. The method employs digital holography to measure the light field at the output end of the six-mode step-index fiber and utilizes the modal orthonormal property of the fundamental mode to calculate the modal coefficients of each mode. Based on the mode distributions measured in Figure 10A1 and A2, the modal weights for all the base modes associated with the patterns are obtained and plotted in Figure 10D1 and D2, and the purity of the specific modes can be obtained. The purities of the OAM_+1_ and OAM_−1_ modes at 1635 nm are 96.95 % and 93.27 %; the OAM_+2_ and OAM_−2_ modes have purities of 90.75 % and 95.81 % at 1548 nm, respectively; at a wavelength of 1460 nm, the OAM_+3_ and OAM_−3_ modes have purities of 96.80 % and 97.03 %, respectively; finally, the purities of the OAM_+4_ and OAM_−4_ modes at 1334 nm are 95.40 % and 95.74 %. Based on the recovered modal weights, the intensity distributions can be reconstructed as presented in Figure 10B1 and B2, and the corresponding phase patterns are shown in Figure 10C1 and C2, respectively. It can be clearly seen that the recovered intensity distributions are highly similar to the measured intensity distributions shown in Figure 10A1 and A2. To quantify the similarity, we calculated the correlation coefficients between the recovered intensity distributions from the modal coefficients and the measured intensity distributions. In the experiments, the correlation coefficients between the recovered and measured mode distributions for the OAM_±1_, OAM_±2_, OAM_±3_, and OAM_±4_ modes are 0.9823 and 0.9772, 0.9655 and 0.9755, 0.9712 and 0.9758, 0.9466 and 0.9512, respectively, which ensures the confidence and accuracy of mode purity. The results demonstrate the successful generation of first- to fourth-order OAM modes with high mode purity. In addition, to evaluate the intermode crosstalk, the power ratios of each component in OAM_±1_, OAM_±2_, OAM_±3_, and OAM_±4_ modes at 1635 nm, 1548 nm, 1460 nm, and 1334 nm, respectively, are shown in Figure 11. The crosstalk of other mode components to the main mode in each OAM mode can be clearly seen. It is worth mentioning that there is no crosstalk caused by the fourth-order mode when measuring the purity of the OAM_±1_, OAM_±2_, OAM_±3_ modes since the transmission of OAM_±4_ modes is not supported at 1635 nm, 1548 nm, and 1460 nm. The results show that first- to fourth-order OAM modes are successfully generated with mode purity higher than 90 % and mode crosstalk lower than −14 dB.

## 4 Conclusions

In summary, the mode converter based on CPT-LPFGs has been proposed to realize the simultaneous generation of first- to fourth-order OAM modes at multiple wavelengths. A segmented Runge–Kutta method is proposed to simulate the preset-twist LPFGs, calculating the twist angle and relative coupling coefficients for each pitch and approximately solving the coupled mode equations, and then comparing the required refractive index modulation at different twist angles. The simulation results indicate that the preset-twist method can effectively increase the coupling coefficients of the higher-order modes and thus reduce the required refractive index modulation to achieve high efficiency. In the experiments, by twisting the fiber at an angle of 1080° and fabricating CPT-LPFGs with periods of 745 μm, 310 μm, 204 μm, and 146 μm, the simultaneous generation of first- to fourth-order OAM modes at wavelengths of 1635 nm, 1548 nm, 1460 nm, and 1334 nm, respectively, has been successfully realized. The insertion loss is less than 1 dB, and the purity of the modes is more than 90 %. This is the first time that a single LPFG is used to generate first- to fourth-order OAM modes simultaneously. The proposed OAM mode converter offers a new approach to achieve a highly integrated multi-order mode converter, with potential applications in the fields of optical communication transmission systems, multi-wavelength vortex lasers and OAM sensing.
